# Anthropometric Indices as Predictors of Coronary Heart Disease Risk: Joint Modeling of Longitudinal Measurements and Time to Event

**Published:** 2017-11

**Authors:** Neda GILANI, Anoshirvan KAZEMNEJAD, Farid ZAYERI, Farzad HADAEGH, Fereidoun AZIZI, Davood KHALILI

**Affiliations:** 1. Dept. of Statistics and Epidemiology, Faculty of Health, Tabriz University of Medical Sciences, Tabriz, Iran; 2. Dept. of Biostatistics, Faculty of Medical Sciences, Tarbiat Modares University, Tehran, Iran; 3. Dept. of Biostatistics, School of Allied Medical Sciences, Shahid Beheshti University of Medical Sciences, Tehran, Iran; 4. Prevention of Metabolic Disorders Research Center, Research Institute for Endocrine Sciences, Shahid Beheshti University of Medical Sciences, Tehran, Iran; 5. Endocrine Research Center, Research Institute for Endocrine Sciences, Shahid Beheshti University of Medical Sciences, Tehran, Iran

**Keywords:** Coronary heart disease, Body mass index, Waist circumference, Waist hip ratio, Height, Joint model

## Abstract

**Background:**

The prevalence of overweight and obesity have increased dramatically worldwide and together they constitute a major risk factor for coronary heart disease (CHD). The aim of this study was to assess the repeated measurements of body mass index (BMI), waist circumference (WC), waist to hip ratio (WHR) and waist to height ratio (WHtR) in predicting CHD incidence.

**Methods:**

This longitudinal study was conducted within the framework of the Tehran Lipid and Glucose Study between 1999–2011, on 1959 women and 1371 men participants’ ages ≥30 yr, without a history of CVD. A joint modeling approach was utilized for data analysis using R software. The resulting joint model allowed measuring α (quantifies the association between anthropometric indices up to time t and the hazard for CHD event at the same time point).

**Results:**

About 9% of the participants (7.1% of the women and 11.7% of the men) experienced CHD event during follow-up. The results indicated a significant linear increasing trend in BMI, WC, WHR, and WHtR over time (*P*<0.001). The increased risk of CHD event in females increases with the values of BMI (α= 0.004, *P*=0.023), WC (α= 0.018, *P*=0.009), WHR (α= 0.067, *P*=0.014) and WHtR (α= 0.106, *P*=0.002). Furthermore, in males the risk of CHD risk increases by the values of BMI (α= 0.005, *P*=0.032), WC (α= 0.019, *P*=0.008), WHR (α= 0.043, *P*=0.015) and WHtR (α= 0.096, *P*=0.002).

**Conclusion:**

By jointly modeling longitudinal data with time-to-event outcomes, our study revealed that WHtR is superior to other indices in predicting CHD incidence.

## Introduction

Based on WHO in 2014, more than 1.9 billion mature persons were overweight, with higher rates among women than in men (1, 2). Over-weight and obesity constitute a major risk factor for coronary heart disease (CHD) are defined clinically as abnormal or excessive accumulation of fat in adipose tissue, to the extent that health is impaired (3, 4). Nevertheless, in relationship with different anthropometric standards regarding obesity, the CHD data is not consistent. There have been some debates in the recent years on the ways and qualities to be used in order to serve as an index on high CHD risk measure in over-weight and obese people (3, 5, 6). The most common anthropometric indices for assessing the weight status are waist circumference (WC), waist to hip ratio (WHR), waist to height ratio (WHtR) and body mass index (BMI). WC, WHR and WHtR have been utilized as measures of abdominal obesity (where visceral adipose tissue is accumulated), and BMI has been used as a measure of general obesity (7).

These surrogate markers are widely employed as predictors of future coronary heart events. The relationship between these indices of obesity with the risk of developing CHD has been well established (5, 8). Most studies on CHD and body weight limit their investigations to BMI, more commonly used in clinical practice. There have been divisions on opinion in taking CHD as a proper forecast measure. Many types of research have suggested applying anthropometric indexes which consider using both abdominals and BMI to predict diseases which would lead to clinical treatment and addresses public health (9–13).

In the past decades, various studies have tried to assess the effects of WC, WHR, WHtR and BMI on the CHD risk, just by applying one measurement for each subject, by the way, these studies are still insufficient in large cohorts (14–16). With respect to these indices which are changing with over time and life styles, thus the regular monitoring of these repeated indices provide more useful information than a single measurement. Typically, classical modeling does not consider dependencies between longitudinal measures of anthropometric indices and CHD incidence. Thus, more complex statistical methods are needed to assess the association between these two types of responses. “A forceful method to overcome this problem is a joint modeling of continuation of life and restated measurements, and so in this large society-based group study” (17).

Therefore, in this large population-based cohort study we utilized a joint modeling of longitudinal measures of anthropometric indices and CHD risk, to know whether these indices can be a significant indicator of predicting CHD incidence.

## Methods

### Study population

This study was performed under the framework of the Tehran Lipid and Glucose Study (TLGS) in triennial examinations. The data from four phases were used conducted in phase I: (1999–2001), phase II: (2002–2005), phase III: (2006–2008) and phase IV: (2009–2011). Concisely, the TLGS is a community-based longitudinal study, performed to explore and prevent non-communicable diseases, in a representative sample of inhabitants, ages > 3 yr, from district 13 of Tehran, the capital city of Iran. The first phase of the TLGS initiated in Mar 1999 to Dec 2001 and data collection, at 3-yr intervals, is continuing. In the first phase, total 15005 individuals in 3-yr old and higher age range were selected at random by using multistage random sampling method. The subjects were all residents in district 13 of Tehran receiving health care in three medical health care centers. The family members, regardless of being in risk factor category or not, were invited to join the measurement baseline with three years’ follow-ups program. All participants were followed for any hospitalized or death event annually up to 20 Mar 2012 (18).

We first considered all participants age≥30 yr and excluded individuals with a history of CVD (women=250 and men=271), leaving us with 4288 women and 3262 men. Of them, 1959 women and 1371 men completed all triennial longitudinal follow-up without missing data with a median 12.4 (interquartile range: 11.9 – 12.7) yr of follow-up.

Informed written consent was obtained from all participants. The study was approved by the Ethics Committee of the Research Institute for Endocrine Sciences, Shahid Beheshti University of Medical Sciences, Tehran, Iran (Ethics code: IR.SBMU.RIES.REC.1394.36).

### Anthropometric, clinical, and laboratory assessments

Details of data collection in TLGS have been published previously (18); in brief, weight was measured to the nearest 100 gr using digital scales while the individuals were minimally clothed, without shoes. Height was measured to the nearest 0.5 cm, in a standing position without shoes, using a tape measure. BMI was calculated as weight (kg) divided by the square of the height (m^2^). WC was recorded to the nearest 0.1 cm at the umbilical level and hip circumference was measured over light clothing at the widest girth of the hip. WHR and WHtR were consequently calculated as the ratios of waist circumference over the hip circumference and waist circumference by height, respectively.

At baseline, some known or suspected risk factors including age, smoking use and laboratory measurements were assessed using previously reported methods. Besides, coronary heart disease, as the main outcome of this study was definite as myocardial infarction, unstable angina pectoris, angiography proven CHD and CHD death. All of them are comparable with ICD10 rubric I20–I25. The event and its corresponding date were confirmed by an outcome committee (18).

### Statistical analysis

The development of dynamic event prediction models that take into accounts both participants’ characteristics and longitudinal anthropometric measurements, requires that we first describe the changes of these indices over time, correcting for baseline variables using linear mixed-effect models. An advantage of the mixed-effects models is that they account for the positive correlation between the measurements observed within the same participant. Different longitudinal sub-models were analyzed with an only intercept, intercept and slope analysis and a non-linear subject-specific evolution for the BMI, WC, WHR and WHtR, separately. Second, survival was studied using a Cox model. The participants’ age (year), sex (1: Male, 2: Female), family history of CHD (1: yes, 0: no), history of tobacco smoking (1: current or past smoker, 0: never smoker), blood pressure and laboratory measurements were included as additional confounders in the survival sub-model. These covariates were added one by one in this sub-model. A covariate was retained in the model if its inclusion improved the log-likelihood significantly (*P*<0.05). Third, because anthropometric indices are time-dependent and not constant between the visits, we considered the joint modeling framework and focused on the assessment of the predictive ability of these indices; all other covariates were considered constant during examination phases. We applied the joint model with shared random-effects method (17, 19). The resulting joint model allowed measuring α (quantifies the association between features of the repeated process up to time t and the hazard for CHD event at the same time point). We proposed a joint model under the maximum-likelihood estimation method. The baseline hazard and the survival function were approximated using penalized B-splines and Gauss-Kronrod quadrature rule, respectively (20). All analyses have been implemented in R-3.2.0, using the JM package (21).

## Results

A total number of 3330 subjects participated including 1959 females and 1371 males. The mean age of females and males were 45.9 (SD 10.6) and 47.6 (SD 12.1) yr at the admission to the study, respectively. There were 17.2% and 19.8% with a history of tobacco use and family history of CVD, respectively.

Table 1 shows the descriptive statistics for anthropometric indices of the sample in four phases. The repeated measures ANOVA test showed a significant increasing trend over time for these anthropometric factors for both gender (*P*<0.001). Regarding the obtained results women’s BMI and WHtR were higher than men’s while men’s WC and WHR were higher than women’s were in each phase.

**Table 1:** Descriptive statistic for anthropometric indices in four phases

***Gender***	***Parameter***	***Phase***				P**
		***1***	***2***	***3***	***4***	
Female	BMI	28.47±4.44^*^	29.42±4.54	29.62±4.60	30.53±4.65	<0.001
	WC	90.23±11.42	93.62±11.38	93.62±11.68	97.84±11.34	<0.001
	WHR	0.86±0.08	0.88±0.08	0.89±0.08	0.95±0.08	<0.001
	WHtR	0.58±0.08	0.60±0.08	0.61±0.08	0.64±0.08	<0.001
Male	BMI	26.45±3.74	26.89±3.75	27.19±3.88	27.35±3.92	<0.001
	WC	90.68±10.49	95.64±10.06	97.01±9.82	98.05±10.34	<0.001
	WHR	0.93±0.07	0.96±0.06	0.98±0.06	0.98±0.06	<0.001
	WHtR	0.54±0.06	0.56±0.06	0.57±0.06	0.58±0.06	<0.001

* mean±SD //

** From repeated measures ANOVA

About 9% of the participants (7.1% of the women and 11.7% of the men) experienced CHD event during follow-up.

**Fig. 1:**
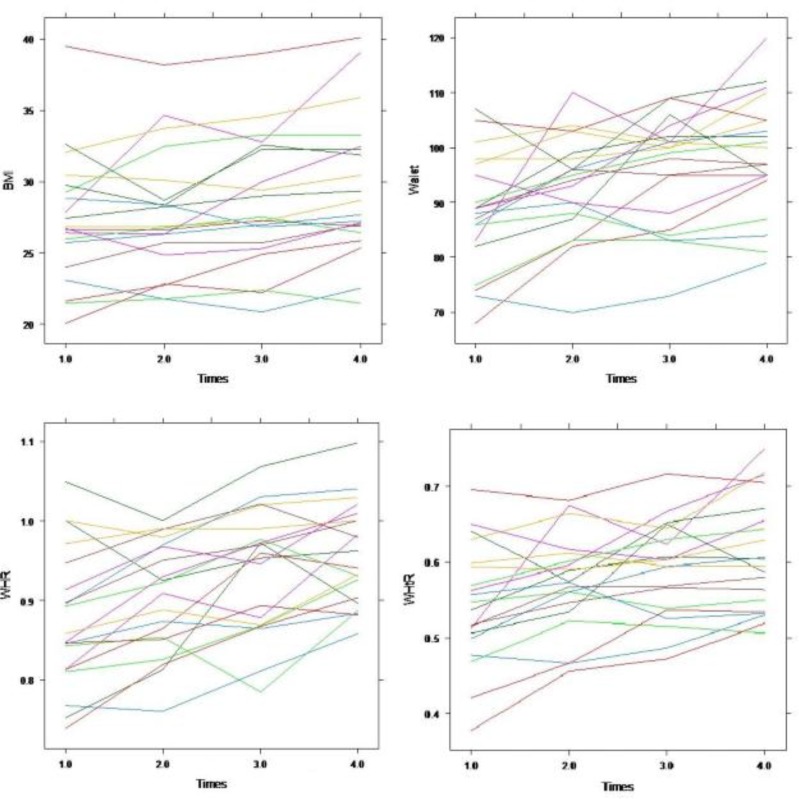
Subject-specific longitudinal trajectories of BMI, WC, WHR and WHtR during the follow-up for twenty random participants

In the first step, we used the ordinary mixed effects and Cox proportional hazard models to select the significant covariates for the longitudinal (BMI, WC, WHR and WHtR) and survival (CHD) outcomes, respectively. Regarding the obtained results from these models (results were not shown in this manuscript) significantly covariates were included in the joint model. Fig. 1 presents the observed trajectories of BMI, WC, WHR and WHtR during the follow-up for twenty randomly selected participants. According to this, the repeated measurements had random intercepts and random trends over time. Therefore, we used random intercept-random time mixed effects models to assess the effect of different factors on these anthropometric indices over time.

In the next step, a joint modeling approach was used for assessing the association between each measure of anthropometric indices and CHD incidence by gender (Tables 2 and 3).

**Table 2:** Results of the joint Modeling of time to CHD event and longitudinal anthropometric indices in females

**Part**	**Covariate**	**Anthropometric Indices**							
		***BMI***		***WC***		***WHR***		***WHtR***	
		***Estimate (SE)***	***P***	***Estimate (SE)***	***P***	***Estimate (SE)***	***P***	***Estimate (SE)***	***P***
Longitudinal	Time	0.624 (0.088)	<0.001	0.911 (0.059)	<0.001	0.029 (0.005)	<0.001	0.017 (0.004)	<0.001
Survival	Age (years)	0.061 (0.010)	<0.001	0.059 (0.010)	<0.001	0.058 (0.010)	<0.001	0.060 (0.010)	<0.001
	Systolic blood pressure, mmHg	0.016 (0.006)	0.006	0.015 (0.006)	0.009	0.014 (0.006)	0.013	0.012(0.008)	0.008
	Diastolic blood pressure, mmHg	0.009 (0.004)	0.003	0.007 (0.001)	<0.001	0.007 (0.004)	0.008	0.008 (0.004)	0.006
	Fasting plasma glucose, mmol/l	0.006 (0.001)	<0.001	0.007 (0.002)	<0.001	0.006 (0.001)	<0.001	0.006 (0.001)	<0.001
	Total cholesterol, mmol/l	0.004 (0.001)	0.011	0.004 (0.002)	0.017	0.004 (0.001)	0.017	0.004 (0.001)	0.015
	HDL cholesterol, mmol/l	−0.024 (0.009)	0.010	−0.025 (0.009)	0.006	−0.026 (0.009)	0.005	−0.025 (0.009)	0.007
	Triglyceride, mmol/l	0.001 (0.001)	0.292	0.001 (0.001)	0.278	0.001 (0.001)	0.228	0.001 (0.001)	0.221
	α	0.004 (0.041)	0.023	0.018 (0.005)	0.009	0.067 (0.168)	0.014	0.106 (0.161)	0.002

**Table 3:** Results of the joint modeling of time to CHD event and longitudinal anthropometric indices in males

***Part***	***Covariate***	***Anthropometric indices***							
		**BMI**		**WC**		**WHR**		**WHtR**	
		**Estimate (SE)**	**P**	**Estimate (SE)**	**P**	**Estimate (SE)**	**P**	**Estimate (SE)**	**P**
Longitudinal	Time	0.307 (0.017)	<0.001	0.348 (0.059)	<0.001	0.017 (0.001)	<0.001	0.014 (0.001)	<0.001
Survival	Age (years)	0.038 (0.008)	<0.001	0.037 (0.008)	<0.001	0.038 (0.008)	<0.001	0.036 (0.008)	<0.001
	Systolic blood pressure (mmHg)	0.016 (0.006)	<0.001	0.010 (0.005)	0.008	0.017 (0.006)	0.006	0.017 (0.006)	0.008
	Diastolic blood pressure(mmHg)	0.009 (0.004)	0.002	0.009 (0.004)	0.002	0.010 (0.004)	0.001	0.009 (0.004)	0.002
	Fasting plasma glucose (mmol/l)	0.007 (0.002)	<0.001	0.009 (0.002)	<0.001	0.007 (0.002)	<0.001	0.007 (0.002)	<0.001
	Total cholesterol (mmol/l)	0.005 (0.002)	0.018	0.005 (0.002)	0.019	0.005 (0.002)	0.020	0.005 (0.002)	0.026
	HDL cholesterol (mmol/l)	−0.006 (0.009)	0.533	−0.006 (0.010)	0.549	−0.006 (0.010)	0.564	−0.007 (0.009)	0.473
	Triglyceride (mmol/l)	−0.001 (0.001)	0.181	−0.001 (0.001)	0.187	−0.001 (0.001)	0.192	−0.001 (0.001)	0.189
	α	0.005 (0.032)	0.031	0.019 (0.004)	0.008	0.043 (0.183)	0.015	0.096 (0.267)	0.002

The variables considered in the survival model were shown in the survival part of the model. The effect of covariates can be interpreted using the model parameters estimates. In the longitudinal part of the model, one can observe a significant effect of times on BMI, WC, WHR, and WHtR during the study period (*P*<0.001). Concurrently the association between these indices and CHD incidence evaluated using the association parameter α. The estimated α indicated significant positive associations between the occurrence of CHD sometimes and required appraise of the anthropometric path along with point; and so, the increased risk of CHD event in females increases with the values of BMI (α= 0.004, *P*=0.023), WC (α= 0.018, *P*=0.009), WHR (α= 0.067, *P*=0.014) and WHtR (α= 0.106, *P*=0.002). Furthermore, in males the risk of CHD risk increases by the values of BMI (α= 0.005, *P*=0.032), WC (α= 0.019, *P*=0.008), WHR (α= 0.043, *P*=0.015) and WHtR (α= 0.096, *P*=0.002).

## Discussion

The joint modeling of longitudinal and time-to-event data is an active field of statistics research that has a lot of notice in the recent years (22–25). A joint model was proposed for assessing the degree of association between the trend of the repeated measurements of aortic gradient and aortic regurgitation and time-to-events of death and reoperation (26). The reason for increased interest is that in focusing either on the longitudinal outcome the joint models can be utilized and we wish to correct for nonrandom dropout or on the survival results when we wish to account for the effect of an endogenous time-dependent endogenous covariate (27).

In our prospective cohort study, with joint modeling approach, BMI, WC, WHR and WHtR were associated with CHD risk in a 12-yrs follow-up period in Tehranian adults. Our findings showed that the relationships between these repeated indices of obesity with the risk of developing CHD were positive, statistically significant, although a higher value of WHtR than other indices increased the risk of CHD (Table 2 and 3). These results are in agreement with the observation that abdominal obesity indicators such as WC, WHR and WHtR, are stronger predictors of CHD risk than general obesity indicator of BMI (28, 29). As per comprehensive review, there was convincing sign that measures of general overweight (e.g. BMI) and measures of abdominal adiposity (e.g. WC, WHR and WHtR) are associated with CVD (5). The most of studies used Classical modeling that did not consider dependencies between the regular monitoring of these repeated indices and CHD incidence. For example, using Cox proportional hazard models concluded that WHtR was statistically the best model fit and strongest associations with CVD (30). In fact, the joint models of longitudinal and survival data indicate a powerful statistical tool capable of capturing the association between longitudinal and survival time data (26). An alternative approach is to utilize the time-dependent Cox model. However, this model assumes a step function between the repeated measurements, which is not realistic for indices because such cardio data as anthropometric measurement values cannot be assumed to be constant between visits. Therefore, in this study we utilized a joint modeling approach, to know whether these indices can be a significant indicator of predicting CHD incidence. BMI was chosen because it is the most widely used index for the evaluation of obesity both in adults and children (31, 32). WC, WHR or WHtR were evaluated because they are is a good correlation with visceral adipose tissue and can better reflect the accumulation of intra-abdominal fat (33, 34). According to longitudinal part of our study, a significant effect of over times in females and males observed on BMI, WC, WHR and WHtR. This relationship on WC in females was more than other indices while in males this relationship on BMI was more other.

Factors such as systolic blood pressure, diastolic blood pressure, fasting plasma glucose, total cholesterol, HDL, triglyceride and time would have their impacts on finding the relationship among the anthropometric indexes on one hand and CHD prevalence on the other hand, we applied the shared/joined modeling in order to assess the conditional effects which are left on CHD occurrence by those predictors. After adjusting for BMI, WC or WHR, the above-mentioned factors still affect the CHD risk. Our findings are consistent with those of other large pooled analyses of prospective cohorts with regard to the positive associations of these indices with CHD (35, 36). Our study showed that after adjusting for blood pressure, fasting plasma glucose and cholesterol, anthropometric measures were still the independent predictors of CHD event which was similar to the finding of pooled analysis of 97 prospective cohorts with 1.8 million participants (37).

In addition, glucose, HDL, cholesterol, triacylglycerols, and diabetes were more strongly associated with WC than with BMI (38).

The present study has a few limitations considered. The fitting of joint models often encounters some difficulties; however, so far there has not been an alternative model universally accepted and serve as a standard statistical advancement to allow joint longitudinal survival model be fit for the research. Furthermore, analyzing large sets of data with joint modeling could be both times consuming and expensive in computational practice. As a prospective cohort study, selection bias may occur due to loss to follow up; this may affect the association of BMI and WC with CHD incidence. We calculated a random intercept effect of follow-up for any participant. In this way, measurement bias was reduced. We do not take into account socioeconomic status and nutritional factors in our analysis. Finally, although participants in TLGS are a good representative of Tehran’s urban population, our findings might not be extrapolated to other parts of Iran, especially its rural areas.

## Conclusion

We utilized a joint modeling approach of the impact of prognostic features on the main endpoints in the trial. In summary, the joint models are a useful tool when longitudinal outcomes are collected together with time-to-event data. Specifically, they incorporate all information simultaneously and provide valid and efficient inferences.

## Ethical considerations

The authors have observed the ethical issues including plagiarism, informing the subjects before taking their consent, refrain from misconduct, data fabrication or falsification, redundancy…) and have committed themselves to abide with those issues.
